# Methylammonium halide salt interfacial modification of perovskite quantum dots/triple-cation perovskites enable efficient solar cells

**DOI:** 10.1038/s41598-023-32697-z

**Published:** 2023-04-03

**Authors:** Ching-Ho Tien, Hong-Ye Lai, Lung-Chien Chen

**Affiliations:** 1 Department of Electronic Engineering, Lunghwa University of Science and Technology, No. 300, Sec. 1, Wanshou Rd., Taoyuan, 33306 Taiwan; 2 Department of Electro-Optical Engineering, National Taipei University of Technology, No. 1, Sec. 3, Chung-Hsiao E. Rd., Taipei, 10608 Taiwan

**Keywords:** Engineering, Materials science, Optics and photonics

## Abstract

Perovskite solar cells (PeSCs) have been introduced as a new photovoltaic device due to their excellent power conversion efficiency (PCE) and low cost. However, due to the limitations of the perovskite film itself, the existence of defects was inevitable, which seriously affects the number and mobility of carriers in perovskite solar cells, thus restricting PeSCs improved efficiency and stability. Interface passivation to improve the stability of perovskite solar cells is an important and effective strategy. Here, we use methylammonium halide salts (MAX, X = Cl, Br, I) to effectively passivate defects at or near the interface of perovskite quantum dots (PeQDs)/triple-cation perovskite films. The MAI passivation layer increased the open circuit voltage of PeQDs/triple-cation PeSC by 63 mV up to 1.04 V, with a high short-circuit current density of 24.6 mA cm^−2^ and a PCE of 20.4%, which demonstrated a significant suppression of interfacial recombination.

## Introduction

Due to the many excellent material properties exhibited by organic–inorganic halide perovskite materials, such as high extinction coefficient, high carrier mobility, and micron-scale carrier diffusion length^1–5^. Perovskite solar cells (PeSCs) have attracted extensive attention in the scientific research community in the past decade and are considered to be very promising photovoltaic materials^6,7^. Its power conversion efficiency (PCE) also rose from 3.8 to 25.7% in just a few years^8^. However, due to the low preparation temperature of perovskite materials and the difficulty in controlling the crystallization process, it was easy to cause a large number of defects on the surface and grain boundaries of the final perovskite film^9,10^, including uncoordinated Pb^2+^, iodine vacancies, Iodine interstitial atoms, lead vacancies and lead-iodine transposition defects, etc. These defects often cause nonradiative recombination and ion migration of carriers, thereby reducing the PCE and long-term stability of the PeSCs^11^.

At present, additives engineering^12^ and interface engineering^13^ were the major methods to reduce defects in passivation perovskite films. In particular, additives engineering can control the crystallization process and passivate defects by introducing passivation substances into the precursor solution, which has the advantages of simple operation and remarkable effect. In the process of realizing the defect passivation, the functional group of the passivator molecule was very important. Carbonyl group^14^, amino group^15^, carboxyl group^16^ and phenethylammonium iodide^17^ passivate defects by forming coordination bonds with unsaturated dangling bonds, thereby prolonging the carrier lifetime and improving device performance parameters. To date, a variety of molecules with different functional groups have been introduced into perovskite precursors as passivating agents. For example, Wang et al.^18^ introduced caffeine into the perovskite precursor, used the strong interaction between caffeine’s C = O and Pb^2+^ to increase the nucleation activation energy, thereby delaying the perovskite nucleation rate and increasing the perovskites quality, the final device obtains 20.25% PCE. Chen et al.^19^ synthesized a π-conjugated and alcohol-soluble small molecule with bilateral carboxyl and thiophene groups, namely 2,5-di(thiophen-2-yl)terephthalic acid (DTA), and added it to the ammonium salt precursor to prepare the perovskite film uses its electron-rich carboxyl groups to form Lewis acid–base adducts with uncoordinated Pb^2+^ in the perovskite film to passivate grain boundaries and surface defects, and finally reduce the device voltage loss to 0.38 V. Although these reported passivator molecules show obvious passivation effect on defects in perovskite films, there were also problems such as complex molecular structure and difficult synthesis. In addition, some surface passivators need to use benzene substances that are harmful to the environment as solvents when they were used for surface treatment of perovskite films, which was not conducive to environmental protection and human health^20^. Therefore, it was still of great significance to find novel passivators with simple structure and environmental friendliness as additives to be introduced into perovskites to prepare high-quality perovskite films and high-performance PeSCs. This work reports an effective passivator for resolving perovskite surface defects, namely methylammonium halide salt, to modify the interface between perovskite quantum dots (PeQDs) film and Cs_0.05_FA_0.81_MA_0.14_PbBr_0.14_I_2.86_ (CsFAMA) triple-cation perovskite film, reducing defects in perovskites. In addition, the use of PeQDs film in the underlying layer of the triple-cation perovskite film can increase the light utilization rate, open circuit voltage (Voc), and short-circuit current density (Jsc), thereby improving the PeQDs/triple-cation PeSCs performance.

## Experimental

### Materials

Nickel nitrate (99.9985%), ethylenediamine (99%), Cs_2_CO_3_ (99.9%), CsI (99.9%), PbI_2_ (99.9985%), and 2,9-Dimethyl-4,7-diphenyl-1,10-phenanthroline (BCP, 98%) were purchased from Alfa Aesar. Ethylene glycol (99%), ethyl acetate (99.8%), hexane (95%), oleylamine (OAm, 80–90%), methylammonium chloride (MACl, 98%), N, N-dimethyl formamide (DMF, 99.5%), dimethyl sulfoxide (DMSO, 99.5%), and toluene (99.5%) were obtained from Echo Chemcial. Octane (99 +%) and 1-Octadecene (ODE, 90%) were purchased from Acros Organics. Methylammonium bromide (MABr, 99.999%) and methylammonium iodide (MAI, 99.999%) were obtained from Luminescence Technology. Oleic acid (OA, 88%), formamidinium iodide (FAI, 99.9%), fullerene-C60 (99.95%), Ag (99.9%), and patterned FTO-coated glass substrates (8 Ω sq^−1^) were obtained from SHOWA, STAREK Scientific, Uni-Onward, Ultimate Materials Technology, and Ruilong.

### Synthesis of CsPbI_3_ perovskite quantum dots

Cs-oleate precursor solution was synthesized by Cs_2_CO_3_ (0.407 g), ODE (20 mL), and OA (1.25 mL) in a 45 mL flask at 120 °C for 30 min under stirring. PbI_2_ (0.5 g) and ODE (25 mL) were stirred in a 4 -mL flask at 120 °C for 30 min. Add preheated (130 °C) OA (2.5 mL) and OAm (2.5 mL) to the PbI_2_-ODE reaction flask until the PbI_2_ was completely dissolved. Then, 2 mL of the Cs-oleate precursor was swiftly injected into the PbI_2_ reaction mixture at 180 °C and then the CsPbI_3_ mixture was quenched into an ice bath. To purify the QDs, EA solution was added to the CsPbI_3_ crude solution with 3:1 in volume ratio and then centrifuged at 6000 rpm for 5 min. The bottom QDs precipitate was added to hexane and EA (1:1 in volume ratio), sonicated for 5 min, and then centrifuged at 6000 rpm for 5 min. Finally, the obtained CsPbI_3_ QDs precipitate was dispersed in 3 mL of octane, then store refrigerated for at least 24 h, and use the supernatant via centrifugation at 6000 rpm for 5 min.

### Device fabrication

**Figure 1:**
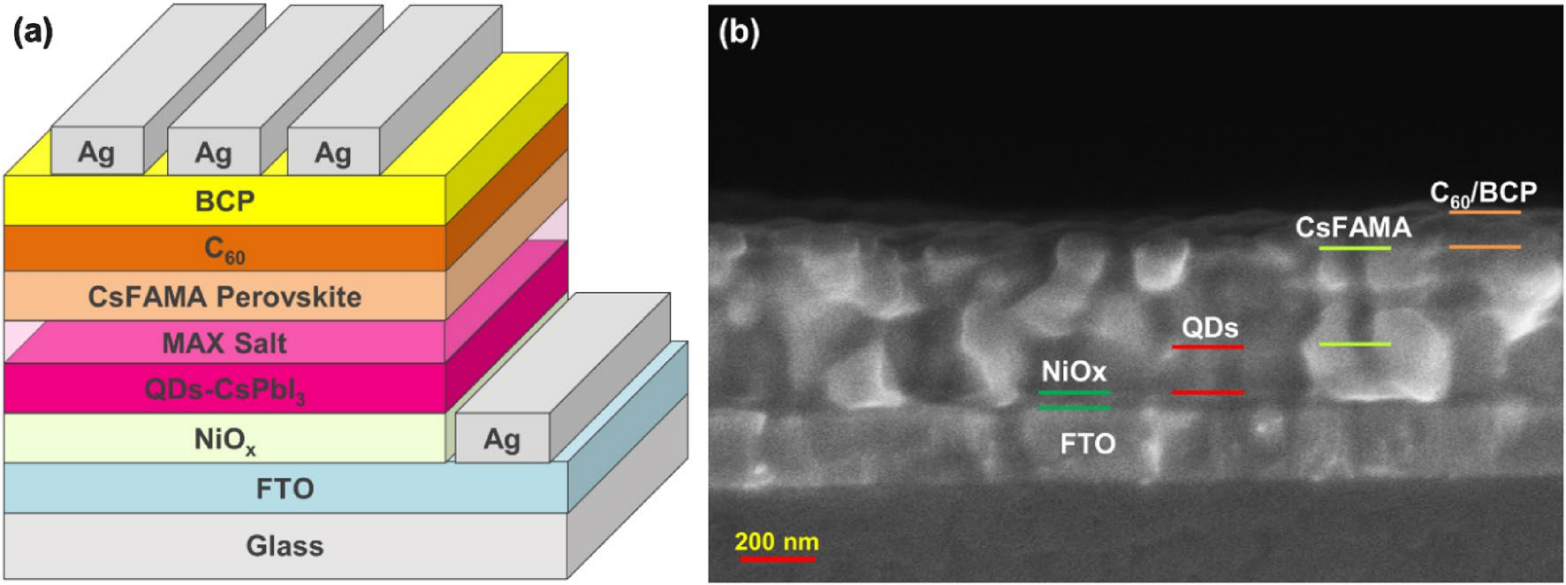
(**a**) Schematic diagram of PeQDs/MAX/CsFAMA PeSC device. (**b**) Cross‐sectional scanning electron microscope (SEM) image.

Patterned FTO anodes were sequentially cleaned by de-ionized water, acetone, ethanol, and isopropanol in an ultrasonic cleaner, and a following ultraviolet ozone treatment for 15 min. of nickel nitrate (0.291 g) was dissolved in ethylenediamine (0.067 mL) and ethylene glycol (1 mL) with magnetic stirring at room temperature for 24 h. The NiOx hole transport layer (HTL) was deposited by spin-coating the filtered NiOx precursor solution on an FTO substrate at 3000 rpm for 30 s and annealed on a hot plate at 120 °C for 10 min, followed by baking in an oven at 300 °C for 1 h. 60 μL of CsPbI_3_ PeQDs solution was uniformly distributed on the NiOx HTL at 1000 rpm for 20 s. 5 mg of methylammonium halide salts (MAX, X = Cl, Br, I) and 5 mL of ethyl acetate were mixed and sonicated for 20 min, and then centrifuged at 6000 rpm for 5 min to obtain the MAX (X = Cl, Br, I) salt solution. Next, 100 μL of MAX salt solution was spin-coated on the CsPbI_3_ PeQDs layer at 2000 rpm for 10 s. Dissolve 461 mg of PbI_2_, 139 mg of FAI, 12.8 mg of CsI, and 15.7 mg of MABr powders in 1 mL of dimethyl sulfoxide (DMSO)/dimethylformamide (DMF) (1/4 vol/vol) at room temperature 25 °C for about 22 h, to prepare the Cs_0.05_FA_0.81_MA_0.14_PbBr_0.14_I_2.86_ triple-cation perovskite precursor solution at a concentration of 1 M. 80 µL of perovskite precursor solution was spin-coated on the PeQDs/MAX layer by a two-step method, using 1000 rpm for 10 s and 5000 rpm for 40 s, respectively. Quickly drop 100 μL of toluene antisolvent onto the PeQDs/MAX layer during the remaining 20 s of the second stage. After the rotation, it was placed in a petri dish for 5 min, and then placed on a hot plate at 100 °C for 10 min to form a Cs_0.05_FA_0.81_MA_0.14_PbBr_0.14_I_2.86_ triple-cation perovskite film. Subsequently, a 20-nm-thick C_60_ electron transport layer and a 5-nm-thick BCP electron blocking layer were sequentially deposited by evaporation under high vacuum. Finally, a 100 nm-thick Ag top electrode was deposited, and its PeQDs/MAX/CsFAMA PeSC structure schematic and cross-sectional SEM image were shown in Fig. 1.

### Characterization

Absorption spectra of perovskite films were tested using a V-770 UV/VIS/NIR spectrophotometer (Jasco, Japan). Use a fluorescence spectrophotometer model Hitachi F-7000 (Tokyo, Japan) to test the photoluminescence (PL) spectrum of perovskite films. X-ray diffraction (XRD) patterns of perovskite films were tested using a PANalytical X’Pert PRO MRD diffractometer (Almelo, The Netherlands) using CuKα (λ = 1.5418 Å) radiation source. The surface morphologies and high-resolution transmission electron microscopy (TEM) image of the perovskite films and PeQDs were observed using a ZEISS Sigma field emission scanning electron microscope (FESEM) instrument (ZEISS, Germany) and a JEM-2100F transmission electron microscope instrument (JEOL, Japan), respectively. The photocurrent–voltage (J–V) curves of the PeQDs/triple-cation PeSCs were obtained by using a MFS-PV-Basic solar simulator (Hong-Ming Technology Co., Ltd., Taiwan) with a Keithley 2420 source meter under illumination of AM 1.5G simulated sunlight at 100 mW cm^−2^, calibrated by a NREL PVM-894 standard silicon reference cell (PV Measurements Inc., USA). External quantum efficiency (EQE) was tested by LSQE-R QE system (LiveStrong Optoelectronics Co., Ltd., Taiwan) equipped with a LAMBDA 35 UV–VIS-NIR spectrophotometer (PerkinElmer, USA) and a XES-204S 150 W xenon lamp (San-Ei Electric Co., Ltd., Japan) as a light source.

## Results and discussion

**Figure 2:**
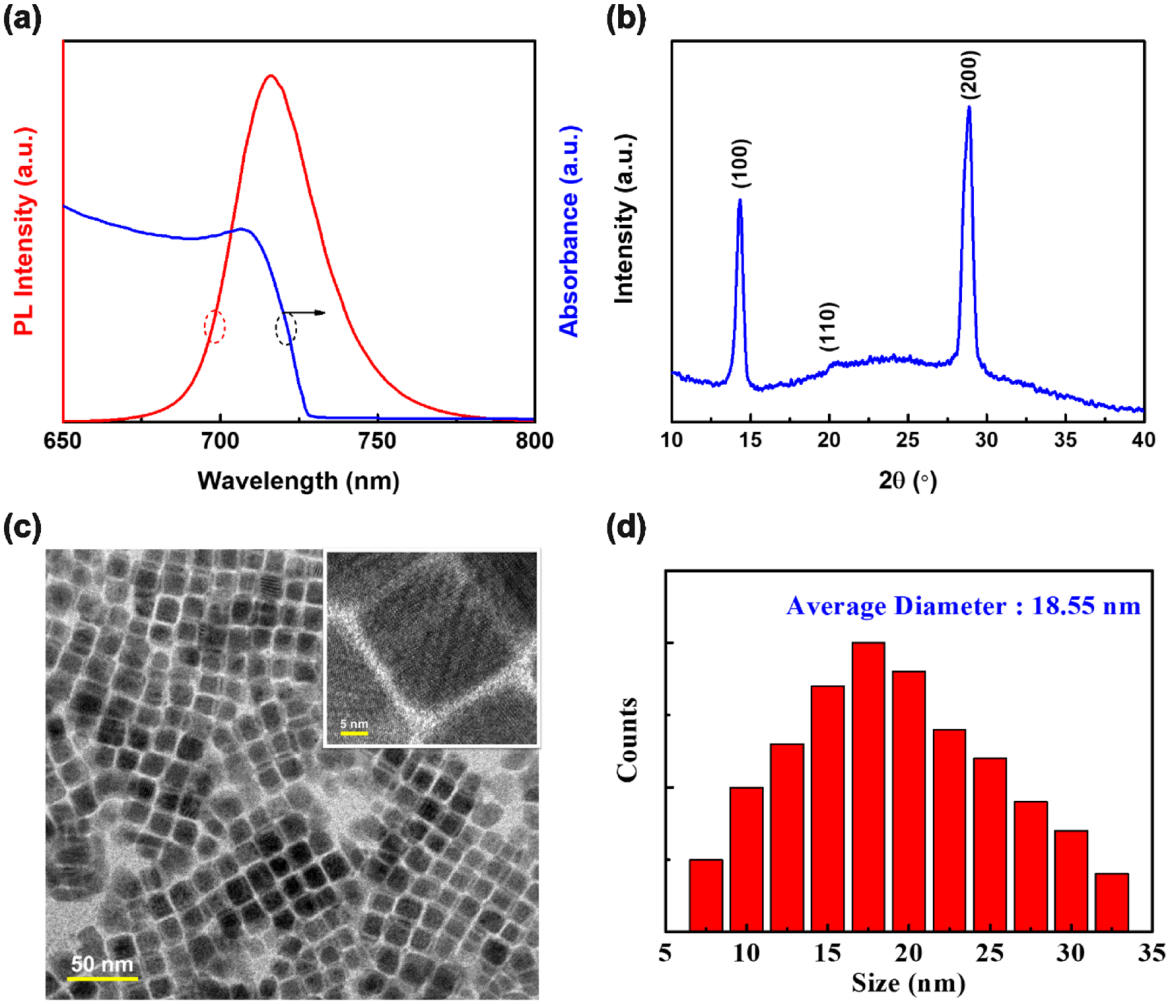
(**a**) Photoluminescence (PL) and absorption spectra, (**b**) XRD pattern, (**c**) TEM image, and (**d**) size distribution chart of CsPbI_3_ PeQDs.

The optical properties and morphology of CsPbI_3_ PeQDs prepared by hot-injection method were characterized. In the absorption spectrum of PeQDs solution in Fig. 2a, it can be found that its absorption peak was about 705 nm, and the corresponding fluorescence spectrum measured the PL peak at 715 nm. Figure 2b depicts the XRD pattern of CsPbI_3_ PeQDs film, indicating that black α-CsPbI_3_ perovskite diffraction peaks appear at 14.33°, 20.23°, and 28.86°, which correspond to the (100), (110), and (200) planes. It was evident from the high-resolution TEM image in Fig. 2c that the as-synthesized CsPbI_3_ PeQDs were in cubic phase with an average size of about 18.6 nm (Fig. 2d).

**Figure 3:**
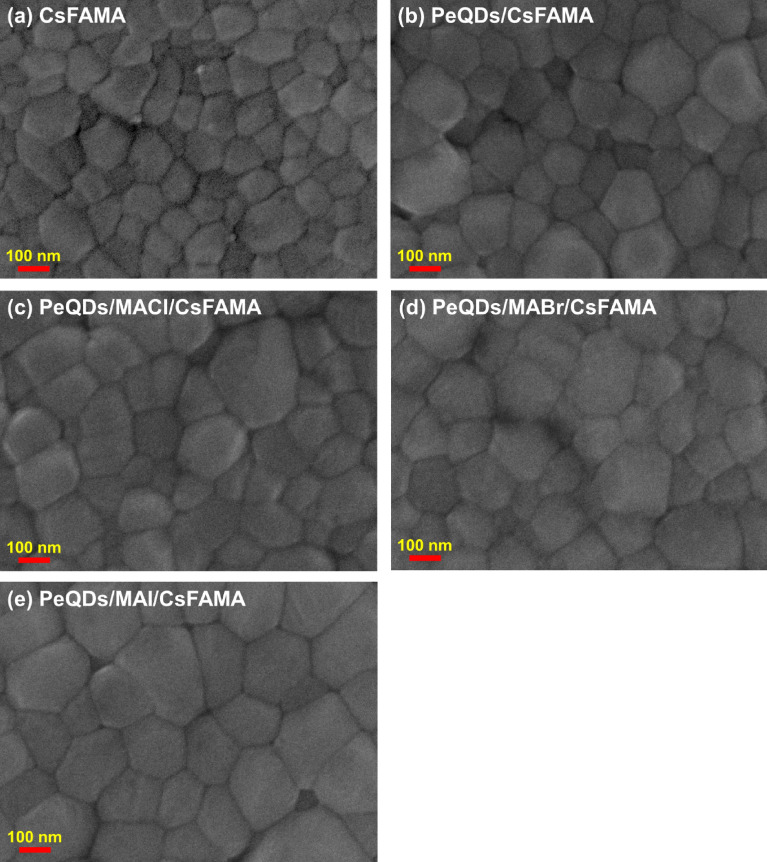
Top view SEM images of the (**a**) CsFAMA, (**b**) PeQDs/CsFAMA, and (**c–e**) PeQDs/MAX (Cl, Br, I)/CsFAMA films.

In order to explore the effect of the introduction of MAX (X = Cl, Br, I) salts on the surface morphology of PeQDs/CsFAMA perovskite films, the above perovskite films were characterized by SEM, as shown in Fig. 3. Figure 3a shows the pure CsFAMA perovskite film, it can be seen that the surface particle size was small and prone to defects and pores. When the CsFAMA perovskite film was covered on the PeQDs film, the particle size of the CsFAMA perovskite becomes larger and the grain boundaries were slightly reduced, as shown in Fig. 3b. Since grain boundaries were one of the main locations for defects, perovskite films with larger grains have fewer grain boundaries, thereby obtaining higher film quality. From Fig. 3c–e, between the introduction of MAX (X = Cl, Br, I) salts to PeQDs/CsFAMA perovskite, it can be observed that the particle size of CsFAMA perovskite increases significantly. The reason for the enlarged perovskite grains may be due to the efficient modification of the PeQDs layer by MAX (X = Cl, Br, I) salts. When CsFAMA perovskites were coated on PeQDs/MAX (X = Cl, Br, I), the rapid nucleation of perovskite may be inhibited and perovskites tend to grow into larger-sized grains at low nucleation density. The results show that the PeQDs/MAI/CsFAMA perovskite has fewer grain boundaries, which can effectively reduce defects. The smooth surface of the perovskite film was beneficial to improve the interface contact between perovskite layer and HTL, and improve the photo-generated charge transfer efficiency^21^.

**Figure 4:**
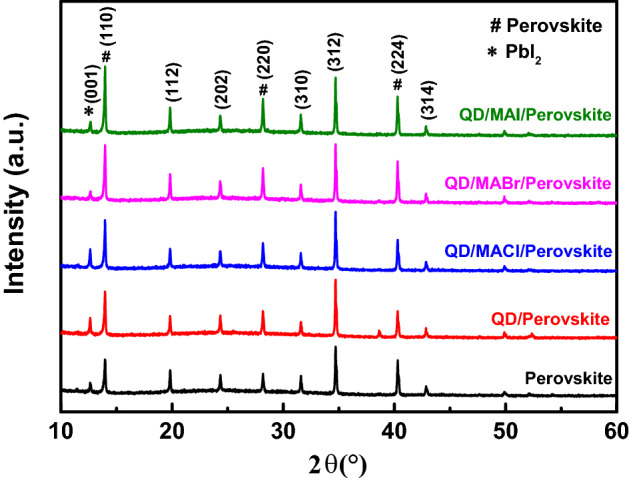
X-ray diffraction (XRD) patterns of the CsFAMA, PeQDs/CsFAMA, and PeQDs/MAX (Cl, Br, I)/CsFAMA films.

Figure 4 shows the X-ray diffraction patterns of CsFAMA, PeQDs/CsFAMA, and PeQDs/MAX (Cl, Br, I)/CsFAMA films, in which the XRD patterns with stronger peaks can represent the formation of crystalline CSFAMA films. The main XRD diffraction peak intensity of the PeQDs/CsFAMA film was higher than that of the pure CsFAMA perovskite film, which indicates that the CsFAMA coating on the PeQDs effectively reduces defects. When the MAX (Cl, Br, I) salts were further introduced into PeQDs/CsFAMA, the XRD intensities of the diffraction peaks of the PeQDs/MAI/CsFAMA films were higher than those of the other two (PeQDs/MACl/CsFAMA and PeQDs/MABr/CsFAMA), representing better film quality. In addition, the non-perovskite β-phase (PbI_2_) peak (001) appeared at 12.7°, it can be found that the PbI_2_ peak intensities of the PeQDs/MAI/CsFAMA and PeQDs/MABr/CsFAMA films were lower, indicating that the formation of PbI_2_ was reduced, which can make it easier for carriers to transition to the HTL, and will not be blocked to cause recombination between carriers. This speculates that the MAX (X = Cl, Br, I) salt diffuses to the grain boundaries and passivates the charge traps at the grain surface and at the grain boundaries, thereby reducing ion diffusion and strengthening bonding by blocking these grain boundary channels.

**Figure 5:**
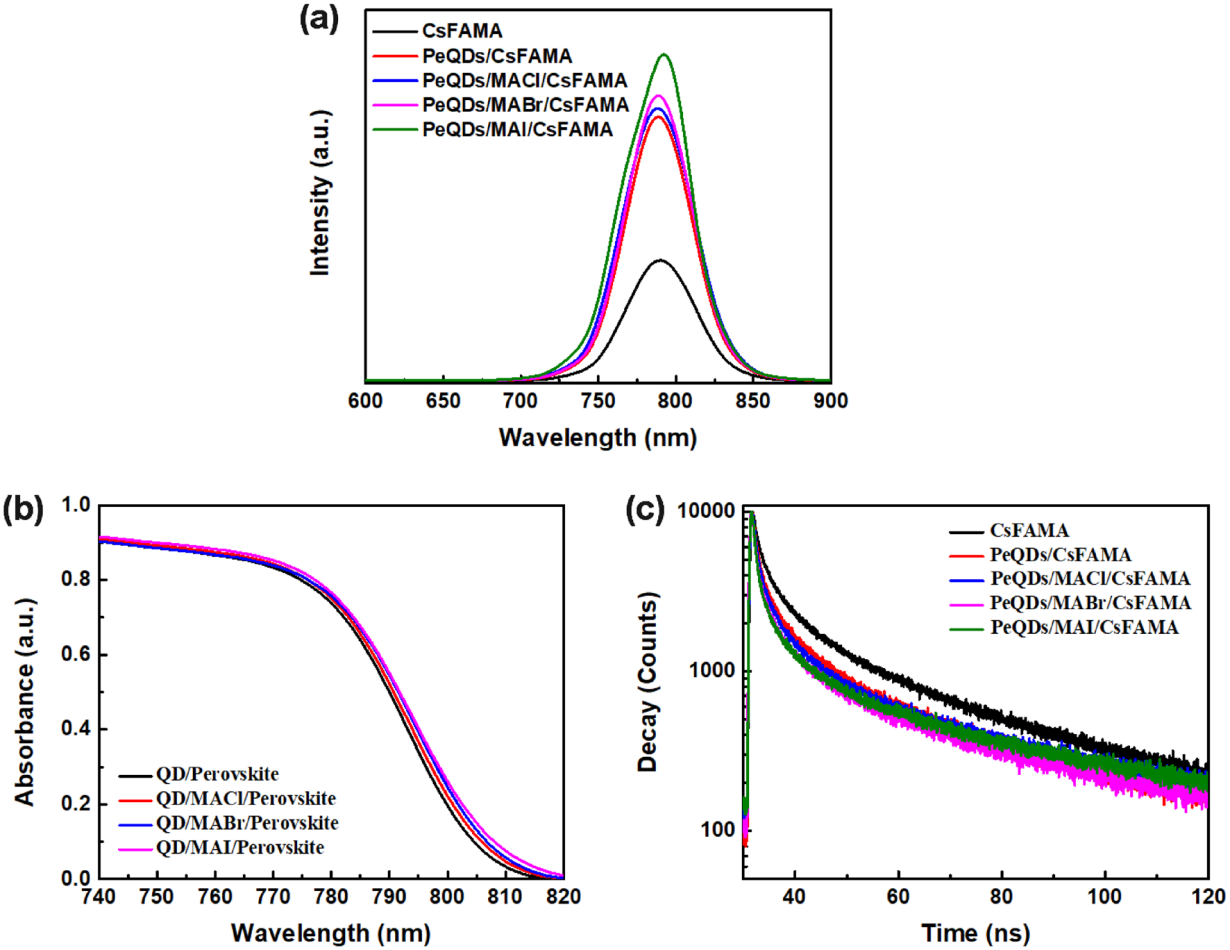
(**a**) Photoluminescence (PL), (**b**) absorption spectra, and (**c**) Time-resolved PL decay curves of the CsFAMA, PeQDs/CsFAMA, and PeQDs/MAX (Cl, Br, I)/CsFAMA perovskite films.

Figure 5a shows the PL spectra of CsFAMA, PeQDs/CsFAMA, and PeQDs/MAX (Cl, Br, I)/CsFAMA films measured under the excitation of wavelength 266 nm. The PL peak of the CsFAMA film was at 790 nm, while the PeQDs/CsFAMA and PeQDs/MAX (Cl, Br, I)/CsFAMA films have a slight blue shift compared to the CsFAMA film. The blue shift may be caused by the disappearance of shallow defect energy level after surface defects were passivated, resulting in an increase in the energy band width^22^. In addition, the defect level accelerates the non-radiative recombination in the perovskite film, resulting in a decrease in the carrier concentration of the perovskite film after reaching steady-state equilibrium under photoexcitation conditions, thereby reducing the radiative recombination rate, i.e. the PL intensity decline. This can also be seen from the intensity of the PL peak. The PL intensity of the CsFAMA film was significantly improved after passivation treatment with PeQDs and MAX (Cl, Br, I) salts. Among them, the PeQDs/MAI/CsFAMA film was the most effective in reducing defects, which proves that the non-radiative recombination in the film was suppressed. The absorption spectra of CsFAMA, PeQDs/CsFAMA, and PeQDs/MAX (Cl, Br, I)/CsFAMA films were shown in Fig. 5b. Compared with CsFAMA perovskite films, PeQDs/CsFAMA and PeQDs/MAX (Cl, Br, I)/CsFAMA passivated perovskite films exhibited slightly enhanced light absorption, which were expected to enhance the EQEs of PeQDs/CsFAMA and PeQDs/MAX (Cl, Br, I)/CsFAMA PeSCs. Because the addition of the PeQDs layer improves the light-harvesting efficiency of the films, and the MAX (Cl, Br, I) salt improves the crystalline quality of the perovskites, the observed performance difference may be attributed to the PeQDs/MAX (Cl, Br, I) Interface engineering leads to more effective charge transfer and increased light absorption across the PSC device. This mechanism can be further demonstrated by the results of the time-resolved PL decay curves of perovskite films. As shown in Fig. 5c and Table 1, the PL decay lifetimes of PeQDs/CsFAMA and PeQDs/MAX(Cl, Br, I)/CsFAMA perovskite films were determined to be 3.92, 3.33, 2.86, and 2.42 ns, while the CsFAMA perovskite film was 5.73 ns. The fast decay lifetimes of PeQDs/CsFAMA and PeQDs/MAX(Cl, Br, I)/CsFAMA perovskite films were significantly lower than that of CsFAMA perovskite film. Since the reduced fast decay lifetime indicates faster and more efficient charge carrier transfer at the interface, it can be concluded that CsFAMA perovskite combined with PeQDs/MAX(Cl, Br, I) has better interfacial properties and charge transfer capacity.

**Table 1:** Time-resolved PL lifetimes of the CsFAMA, PeQDs/CsFAMA, and PeQDs/MAX (Cl, Br, I)/CsFAMA perovskite films.

Sample	A_1_ (%)	τ_1_ (ns)	A_2_ (%)	τ_2_ (ns)	τ_av_ (ns)
CsMAFA	74.6	1.8	25.4	17.2	5.73
PeQDs/CsMAFA	80.8	1.3	19.2	14.6	3.92
PeQDs/MACl/CsMAFA	83.3	53.7	16.7	13.7	3.33
PeQDs/MABr/CsMAFA	85.9	1.1	14.1	13.3	2.86
PeQDs/MAI/CsMAFA	87.9	1.0	12.1	12.4	2.42

**Figure 6:**
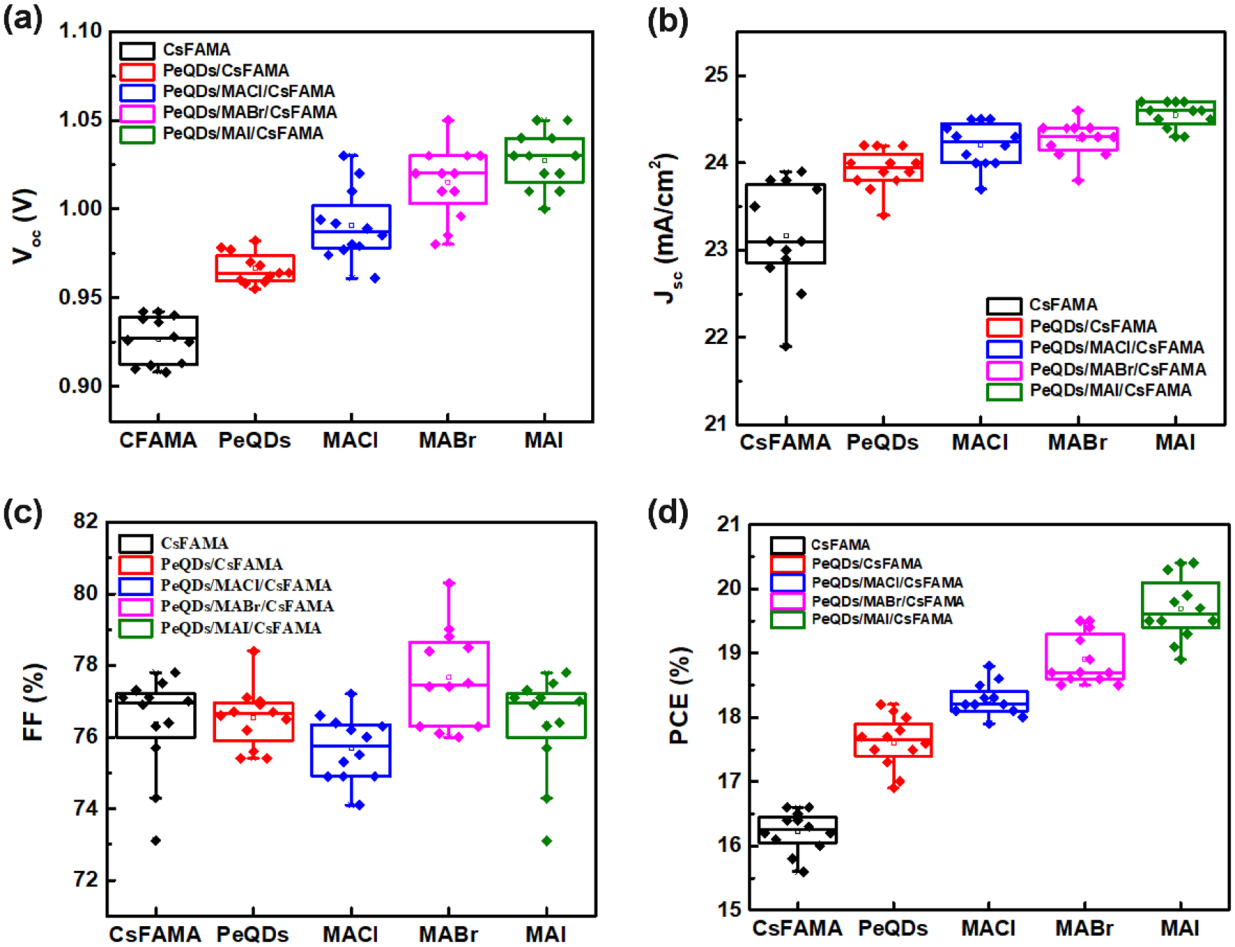
Statistical distribution of (**a**) Voc, (**b**) Jsc, (**c**) FF, and (**d**) PCE of the PeSCs based on the CsFAMA, PeQDs/CsFAMA, and PeQDs/MAX (Cl, Br, I)/CsFAMA.

**Figure 7:**
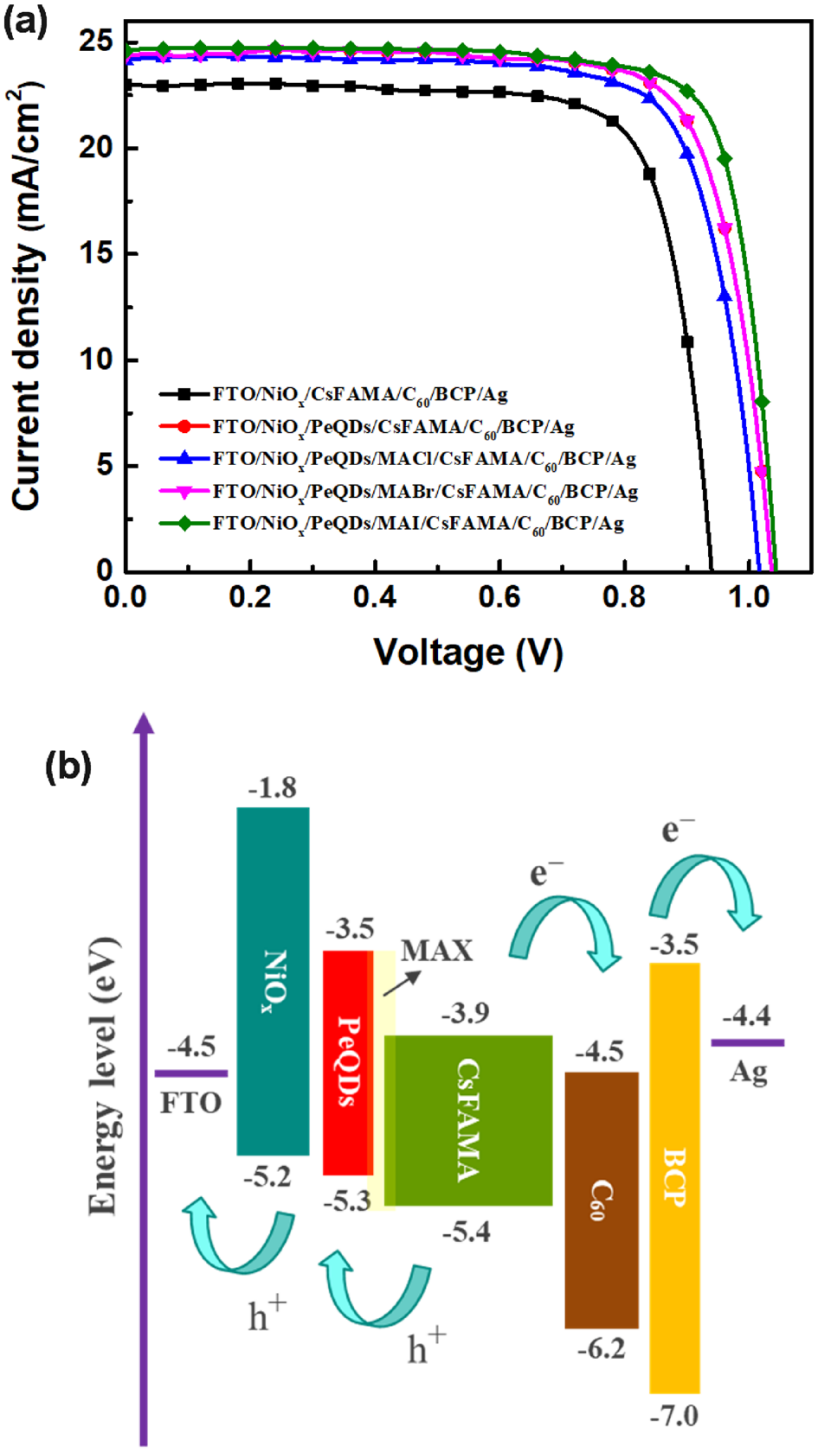
(**a**) J–V curves of the best-performing PeSCs based on the CsFAMA, PeQDs/CsFAMA, and PeQDs/MAX (Cl, Br, I)/CsFAMA. (**b**) Band energy diagram of the whole structure.

To further explore the effect of introducing MAX (X = Cl, Br, I) salts on the photovoltaic performance of PeSCs, and to evaluate the reproducibility of PeSCs, more than 30 cells were fabricated and tested in each case. Figure 6 shows the statistical results of photovoltaic parameters of the PeSCs based on the CsFAMA, PeQDs/CsFAMA, and PeQDs/MAX (Cl, Br, I)/CsFAMA. The device of pristine CSFAMA PeSC had an averaged Jsc of 23.1 mA cm^−2^, a Voc of 0.93 V, a fill factor (FF) of 76.9%, and a PCE of 16.2%. After treating by MAI salt, the parameters were enhanced to an averaged Jsc of 24.6 mA cm^−2^, a Voc of 1.03 V, an FF of 77.8%, and a PCE of 19.6%. The average photovoltaic properties of all other PeSCs were also better than the pristine CSFAMA PeSC, indicating the reliability of the testing results. This indicates that the introduction of MAX (X = Cl, Br, I) salts were believed to play an important role in improving photovoltaic parameters. Figure 7 shows the J-V curves of the best-performing PeSCs in each case, and the corresponding photovoltaic performance parameters were summarized in Table 2. The PeSC based on pristine CsFAMA gave a PCE of 16.6% with a Jsc of 23 mA cm^−2^, a Voc of 0.94 V, and a FF of 77.1%. When the PeQDs layer was introduced into the CsFAMA PeSC, its Jsc, Voc, FF and PCE were significantly increased to 24.2 mA/cm^−2^, 0.977 V, 76.7% and 18.1%, which indicated that adding the PeQDs layer could improve the quality of the CsFAMA film and at the same time increase the photocurrent. When MAX (X = Cl, Br, I) salts modified the PeQDs/CsMAFA interface, the optimized PeSC fabricated with MAI-treated PeQDs/CsMAFA perovskite exhibited an increased Jsc of 24.6 mA cm^−2^, a Voc of 1.04 V, an FF of 79.9%, subsequently, an enhanced PCE of 20.4%. On the other hand, it can be observed that the FF of PeQDs/CsFAMA and PeQDs/MACl/CsFAMA PeSCs were lower than that of the pristine CsFAMA PeSC, which may be caused by the non-perovskite β-phase peak. Additionally, the devices based on MACl- and MABr- treated PeQDs/CsMAFA interface just gave the PCEs of 18.8% and 19.5%, respectively, validating the positive effects of this surface passivation process. The positive effect of this surface passivation process to effectively reduce defects was verified by modifying the PeQDs/CsMAFA interface with MAX (X = Cl, Br, I) salts. Figure 7b illustrates the transfer of photogenerated electrons and holes when PeQDs was inserted into the interface between the perovskite layer and the HTL. Since the valence band position of PeQDs was located between HTL and perovskite, coupled with the passivation of MAX (X = Cl, Br, I) salts, a more favorable energy level alignment occurs at the interface to facilitate hole extraction. The PeQDs acted as a barrier to reduce the carrier recombination at the interface between the perovskite and HTL, thereby improving Voc and Jsc, and preventing the backflow of electrons from the conduction band of the perovskite to the HTL, thereby improving charge collection.

**Table 2:** Photovoltaic characteristics of the best-performing PeSCs based on the CsFAMA, PeQDs/CsFAMA, and PeQDs/MAX (Cl, Br, I)/CsFAMA.

Sample	Voc (V)	Jsc (mA cm^−2^)	FF (%)	PCE (%)
CsMAFA	0.938	23.0	77.1	16.6
PeQDs/CsMAFA	0.977	24.2	76.7	18.1
PeQDs/MACl/CsMAFA	1.02	24.1	76.3	18.8
PeQDs/MABr/CsMAFA	1.03	24.4	77.8	19.5
PeQDs/MAI/CsMAFA	1.04	24.6	79.9	20.4

**Figure 8:**
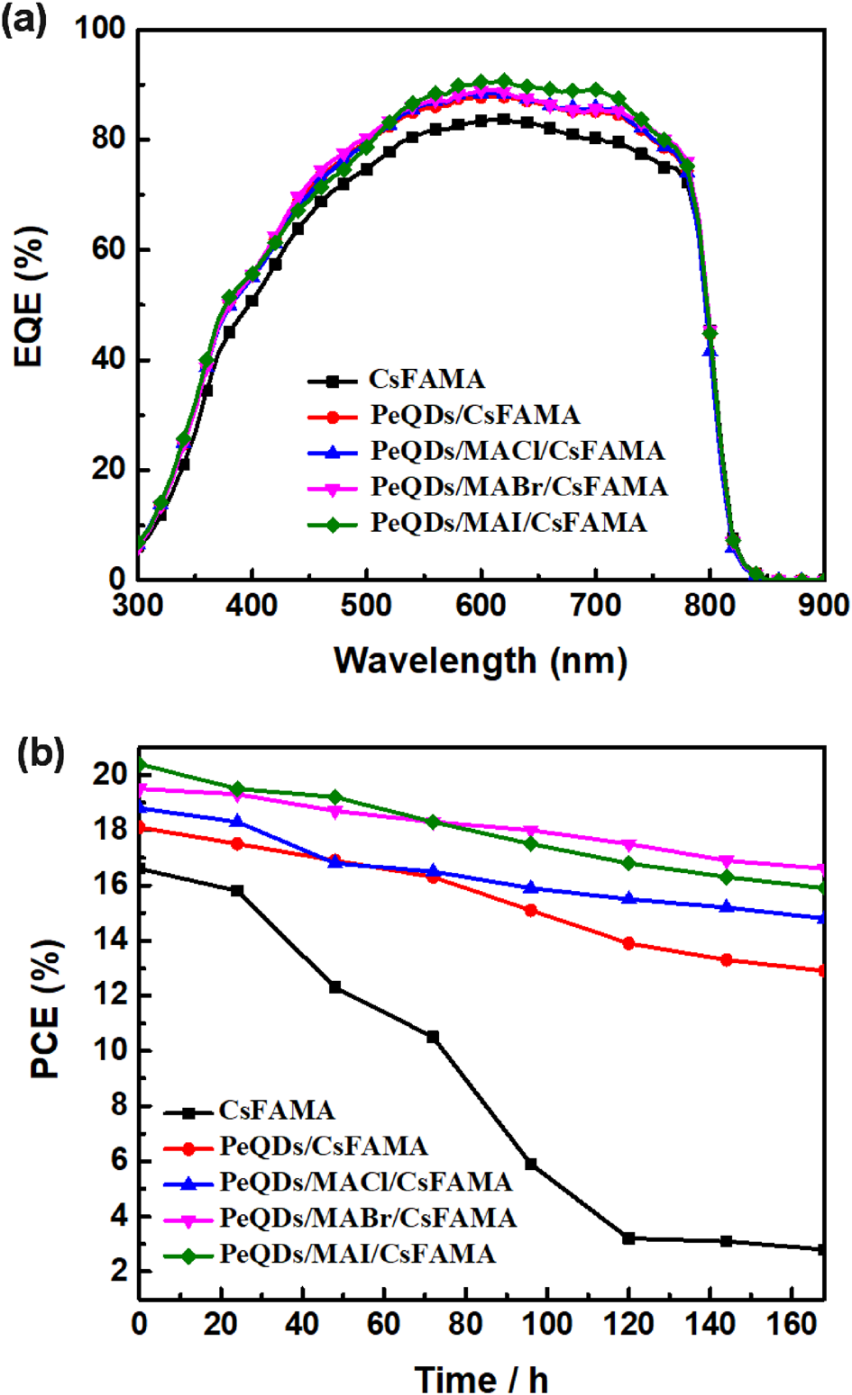
(**a**) External quantum efficiency (EQE) spectra and (**b**) normalized PCE stability evolution (N_2_-filled glovebox) of the PeSCs based on the CsFAMA, PeQDs/CsFAMA, and PeQDs/MAX (Cl, Br, I)/CsFAMA.

Figure 8a shows the EQE spectra of the PeSCs based on the CsFAMA, PeQDs/CsFAMA, and PeQDs/MAX (Cl, Br, I)/CsFAMA. It can be seen that adding a PeQDs layer to CsFAMA PeSC can effectively increase its Jsc and EQE, and there was a relatively obvious increase in the band of 500–750 nm. Through the quantum confinement effect, the absorption generated by PeQDs occurs in this band. Furthermore, the modification/passivation of the interface between PeQDs and CsFAMA using MAX (X = Cl, Br, I) salts further improve the film crystallinity and reduce defects. The quantum efficiency of the display device in this wavelength range is greatly affected by the interface behind the light-absorbing layer. This phenomenon shows that the carrier recombination at this interface is significantly suppressed after passivation, and it also explains the improvement mechanism of the short-circuit current density of the device by passivation. To investigate the effect of MAX (X = Cl, Br, I) salts on the stability of PeSCs, the PCE decays of MAX salt-treated PeSCs and control CsFAMA PeSCs were tracked and recorded at 25 °C in nitrogen storage and under AM1.5G illumination. The PCE loss of CsFAMA PeSC was more than 80% after 120 h storage. In addition, the addition of the PeQDs layer to PeQDs/CsFAMA PeSC effectively reduced defects and greatly improved the phenomenon of PCE attenuation. The PeSCs treated with MAX (X = Cl, Br, I) salts retained 78.7%, 85.1% and 77.9% of their initial efficiencies, respectively (Fig. 8b). On the other hand, the reason for the more attenuation of PeQDs/MAI/CsFAMA PeSCs were that the bonding between iodide ions and organic cations were weak. After several days of exposure, the non-perovskite β-phase peak appeared, leading to the decomposition of perovskites and reducing the stability.

## Conclusion

In summary, this study uses methylammonium halide salt (MAX, X = Cl, Br, I) as a modifier/passivator for the interlayer of PeQDs and triple-cation perovskites. CsPbI_3_ PeQDs and Cs_0.05_FA_0.81_MA_0.14_PbBr_0.14_I_2.86_ triple-cation perovskites and solar cell devices were prepared by hot injection and two-step methods. The effect of methylammonium halide salt on the morphology, optical properties of perovskites and device performance of PeQDs/MAX/triple-cation PeSCs were investigated. The results show that MAX salt can modify/passivate the interaction between PeQDs/CsFAMA perovskite to increase the perovskite grain size and effectively reduce the defects of perovskite films. Compared with the control sample of pure CsFAMA, the best performance of PeSCs was achieved by using MAI salt, the Jsc of PeQDs/MAI/CsFAMA triple-cation PeSCs prepared under this condition increased from 23.0 mA cm^−2^ to 24.6 mA cm^−2^, Voc from 0.938 V to 1.04 V, FF from 77.1% to 79.9%, and PCE from 16.6% to 20.4%.

## Data Availability

The datasets used and/or analyzed during the current study available from the corresponding author on reasonable request.
